# The role of lung ultrasound and ultrasound elastography in diagnosis of interstitial lung diseases

**DOI:** 10.5578/tt.202402873

**Published:** 2024-06-12

**Authors:** Zarifa ABDULLAYEVA, Müge AYDOĞDU, Ebru ÖZTÜRK, Nilgün YILMAZ DEMİRCİ, Haluk Şaban TÜRKTAŞ, Nurdan KÖKTÜRK

**Affiliations:** 1 Department of Pulmonary Diseases, Gazi University Faculty of Medicine, Ankara, Türkiye; 2 Department of Biostatistics, Hacettepe University Medical Sciences Institute, Ankara, Türkiye

## Abstract

**ABSTRACT**

**
The role of lung ultrasound and ultrasound elastography in diagnosis of interstitial lung diseases
**

**Introduction:** *Ultrasound elastography (US-E) is a novel, tissue stiffness-sensi- tive imaging method. We aimed to investigate whether lung ultrasound (US) and US-E can play a role in diagnosing interstitial lung diseases (ILDs) in which lung elasticity is affected due to fibrosis.
*

**Materials and Methods:** *A prospective cohort study. Patients with ILD were defined as ‘‘ILD group’’ and with other pulmonary diseases as ‘‘control group”. All subjects were examined and compared by lung US in B and elastography modes. Besides, the relationship between ultrasonography and high-resolution computerized tomography (HRCT) and chest X-ray findings was evaluated.
*

**Results:** *A total of 109 patients, 55 in ILD and 54 in the control group, with a mean age of 62 ± 14 years, were included. A positive correlation was found between the Warrick score (calculated from HRCT to determine the severity of ILD) and the number of B-lines (discrete vertical reverberation artifacts, indicating interstitial lung syndrome) in lung US (p= 0.001, r= 0.550) in the ILD group. In US-E, blue color (meaning more rigid tissue) dominated in the ILD group, and green color (indicating medium tissue stiffness) dominated in the control group (p= 0.001). Lung US diagnosed the ILD with 69% accuracy, 80% sensitivity, and 60% specificity compared to HRCT. Combined with chest X-ray, diagnostic accuracy was 74%, sensitivity 60%, and specificity 89%.
*

**Conclusion:** *Although lung US and US-E are not superior to gold standard HRCT in diagnosing ILDs, they can still be accepted as promising, novel, non- invasive tools, especially when combined with chest X-rays. Their role still needs to be clarified with further studies.
*

**Key words:** *Lung; tomography; X-ray; ultrasonography; fibrosis; elasticity imaging techniques
*

**ÖZ**

**
İnterstisyel akciğer hastalıklarının tanısında akciğer ultrasonu ve ultrason elastografinin yeri
**

**Giriş:** *Ultrason elastografi (US-E) yeni, doku sertliğine duyarlı bir görüntüleme yöntemidir. Biz bu çalışmada, akciğer ultrasonunun (US) ve US-E’nin, fibrozis nedeniyle akciğer elastikiyetinin etkilendiği interstisyel akciğer hastalıklarının (İAH) tanısındaki rolünü araştırmayı amaçladık.
*

**Materyal ve Metod:** *Prospektif bir kohort çalışması. Daha önce interstisyel akciğer hastalığı tanısı almış veya İAH ile uyumlu bulgula- rı olan hastalar “İAH grubu”, diğer akciğer hastalıkları olan hastalar ise “kontrol grubu” olarak tanımlandı. Her iki grup da akciğer US ile B mod ve elastografi modunda incelendi ve karşılaştırıldı. Ayrıca ultrasonografi ile yüksek çözünürlüklü bilgisayarlı tomografi (YÇBT) ve akciğer grafisi bulguları arasındaki ilişki değerlendirildi.
*

**Bulgular:** *Çalışmaya yaş ortalaması 62 ± 14 yıl olan 55’i İAH, 54’ü kontrol grubunda olmak üzere toplam 109 hasta dahil edildi. İnterstisyel akciğer hastalığı grubunda Warrick skoru (ILD’nin ciddiyetini belirlemek için YRBT’den hesaplanmıştır) ile akciğer US’deki B çizgilerinin sayısı (interstisyel akciğer sendromunu gösteren ayrık dikey yankılanma artefaktları) arasında pozitif bir korelasyon bulundu (p= 0,001, r= 0,550). US-E’de İAH grubunda mavi renk (sert doku), kontrol grubunda ise yeşil renk (orta sertlikte doku) hakimdi (p= 0,001). Akciğer US’u, İAH’yı YRBT’ye kıyasla %69 doğruluk, %80 duyarlılık ve %60 özgüllükle teşhis etti. Akciğer gra- fisi ile birleştirildiğinde tanısal doğruluk %74, duyarlılık %60 ve özgüllük %89 idi.
*

**Sonuç:** *Her ne kadar akciğer US ve US-E, İAH tanısı koymada altın standart YRBT’ye üstün olmasa da, özellikle göğüs röntgeni ile birleştirildiğinde umut verici, yeni, invazif olmayan araçlar olarak kabul edilebilir. Rollerinin daha ileri çalışmalarla açıklığa kavuşturul- ması gerekmektedir.
*

**Anahtar kelimeler:** *Akciğer; tomografi; X-ray; ultrason; fibrozis; elastisite görüntüleme yöntemleri
*

## INTRODUCTION

Interstitial lung diseases (ILD) are characterized by diffuse interstitial damage and impaired alveolar- capillary membrane gas exchange capacity due to fibrosis (1-3). Diagnosing pulmonary fibrosis could be difficult, particularly in the early stages of the dis- ease, due to non-specific symptoms. High-resolution computerized tomography (HRCT) is the gold stand- ard imaging technique in diagnosing and following ILDs (1-3). However, using serial HRCTs at the time of diagnosis and during follow-up periods leads to increased cost and high radiation exposure.

Lung ultrasonography (US) is increasingly used for diagnosing and treating pleural diseases, particularly in atelectasis and consolidation of the lungs (4-7). Besides, it has been recently used to assess interstitial lung diseases (8-10). “B mode” US provides morphologic imaging of the organs while ultrasound elastography (US-E) provides quantitative and qualitative information about tissue elastic properties, which may be predominant in ILDs (11,12). To better understand fibrotic tissue in ILDs, elastography features could be added to lung US (11). It has been further developed and refined recently to enable quantitative assessments of tissue stiffness in ILDs (12-15).

US-E measures tissue stiffness using strain elastography and shear wave elastography. Strain elastography uses a color map to indicate soft,

medium, and hard tissue, called an “elastogram”. The red/yellow color indicates soft tissue, the green color indicates medium hardness, and the blue color indicates more rigid tissues. Tissue stiffness can be measured using a semi-quantitative strain ratio, calculated by selecting the region of interest (ROI) in normal and pathological tissue (12-15). The more rigid the tissue, the higher the strain ratio will be. On the other hand, shear wave elastography is quantitative, and an acoustic push pulse is used to measure tissue stiffness. This is called an “acoustic radiation force impulse” (ARF) (12-15). Studies with elastography have shown that this method can differentiate many solid tumors from healthy tissues (16,17). Few studies for ILDs have used shear wave elastography and revealed that US-E is a safe, non- invasive technique with high sensitivity for detecting ILDs (18-21). However, there is no study that has used color scale elastography and strain ratio measurement to diagnose ILDs and determine the severity of the disease.

Therefore, we hypothesized that lung US and color scale US-E may be crucial and helpful for screening and early diagnosis of ILDs, and they may also be used to assess disease severity.

### MATERIALS and METHODS

The study was conducted at the Pulmonary Diseases Department of a university hospital from September 2018 to April 2019. Local Ethics Committee approved

the research on 26.02.2018 with decision number 149, and participants signed an informed consent form.

### Participants and Control Subjects

Participants over 18 years of age who were followed up with a definite diagnosis of ILD and who were primarily considered to have ILD were included in the study as the “ILD group” and the ones who were followed up in the pulmonary ward for any non-ILD causes were included as the ‘‘control group’’. ILD diagnosis was based on history, clinical findings (progressive dyspnea, chronic cough), examination, and laboratory and instrumental tests, including pulmonary function tests (restrictive pattern), lung histopathology, and HRCT. All patients were diagnosed with ILD (idiopathic pulmonary fibrosis or other idiopathic interstitial pneumonia) according to the guidelines (1-3). The control group comprised patients admitted to the pulmonary ward for diseases other than ILD. The patients who had refused to participate in the study and had cardiogenic and non-cardiogenic pulmonary edema were excluded.

Demographic characteristics of the patients (age, sex, smoking history, and occupation), additional diseases, and exposure histories were questioned and recorded.

### Diagnostic Measurements

The patients were evaluated in the pulmonary ward with physical examination findings and with diagnostic tests such as spirometry measurements

[FVC (mL and %), FEV1/FVC], lung volumes (TLC) and diffusion (DLCO) tests, 6-minute walking test,

connective tissue markers, arterial blood gas values, PA chest X-ray, HRCT findings and echocardiography (pulmonary arterial pressure values). The final diagnoses and treatment information were also noted.

High-resolution computed tomography sections were evaluated during hospitalization. The modified Warrick score was used in the assessment. Warrick score is a scoring method that assesses the severity and extension of parenchymal pathology in patients with ILDs (22). The total score can range from 0-30; an increase in the score indicates an increase in the degree of pulmonary involvement (Table 1).

Images compatible with emphysema, pulmonary nodule, mass, traction bronchiectasis, mosaic attenuation and consolidation at HRCT, lung US, and chest X-ray were also noted.

### Lung US Evaluation

Participants were examined using the “Hitachi, Hi-Vision Avius (Hitachi Aloka Medical, Tokyo, Japan)” ultrasound device. For performing lung US and US-E, the “HITACHI EUP-C532 Micro Convex” probe with a frequency rate of 4-8 MHz was used at the abdomen preset. Depth was adjusted according to the patient. In obese patients or patients with large muscles, depth was increased, and it was lowered in thin patients. Approximately, depth was adjusted between 8-14 cm. Tissue harmonic imaging was turned off, and time gain compensation was kept midline for most image acquisitions. However, gain compensation was increased for some patients for better image provision. The strain elastography method was used since the ultrasound device and elastography probe were incompatible with shear wave elastography.

The patients were examined in both sitting and lying down positions. Both lungs were evaluated through intercostal spaces with a comprehensive US assessment method (10). For the anterior and lateral regions of the right chest wall, the lung US and US-E

were performed by evaluating the 2nd to 5th intercostal

spaces on the parasternal, midclavicular, anterior

**Table:** 

**Table 1.** Warrick score			
**Severity Score** **Abnormality**	**Score**	**Extension Score** Bronchopulmonary segment*	**Score**
Ground glass opacity	1	1-3 segment	1
Pleural irregularity	2	4-9 segment	2
Septal/sub pleural lines	3	>9 segment	3
Honey combing	4		
Sub pleural cyst	5		
Maximum severity score	15	Maximum extension score	15
*For each abnormality, the affected number of segments is scored.

axillary, and mid-axillary lines. The 2nd to 4th intercostal spaces were assessed along the same lines for the anterior left chest wall. The left 5th intercostal space was not evaluated since the heart blocks correct visibility. Thus, a total of 28 intercostal spaces were evaluated antero-laterally. At the posterior chest wall, the 7th and 8th intercostal spaces on posterior axillary and subscapular lines and the 2nd to 8th intercostal spaces on the paravertebral lines were assessed, totaling 22 bilaterally. Hence, 50 intercostal spaces were evaluated in both lungs in the anterolateral and posterior chest wall, both in B and elastography modes (10). The structures that were assessed during lung US were defined as follows (Figure 1A-D).

Pleural irregularities were evaluated, and pleural thickness was measured in B mode.

“A” lines were defined as horizontal hyperechoic lines below and parallel to the pleural line and associated with lung sliding (to and fro dynamic lung movement during respiration visible at the pleural line) (4,7,8).

“B” lines were defined as discrete laser-like vertical hyperechoic reverberation artifacts that arise from the pleural line, extend to the bottom of the screen without fading, and move synchronously with respiration (4,7,8). Multiple B line artifacts were defined as the sonographic sign of a so-called “interstitial lung syndrome” (7-9). B-lines are visible when the lung parenchyma air content is partially decreased, and the interstitial space is volumetrically expanded, such as in pulmonary edema of various etiologies and interstitial lung disease (7-9). The number of B lines in each area was noted. The total number of B lines was recorded. The presence of more than 5B lines between the two levels was considered “B positive.”

### Lung US-elastography Evaluation

While performing the US, B mode images on the right and elastography images on the left were examined simultaneously. To perform strain elastography evaluation, slight compression movements were conducted from the intercostal space to the thorax, and the patient was told to “breathe in and out deeply” or “breathe in deeply and hold his breath” (11-14). An elastography image could not be obtained in all areas. When it was received, the image was frozen. Color intensity (red, green, blue) was evaluated. Suitable regions were determined for strain ratio measurement. First, ROI (“A”) was selected from the green or red colored area close to the pleura as a reference tissue, then ROI (“B”) was chosen from the blue area where the B lines are intense, or from the random blue area close to the pleura if there is no B line (11-14). The strain ratio is calculated using the B/A formula (Figures 2 A,B).

Three different observers evaluated the lung US and US-E (Aydoğdu M, Demirci NY, Abdullayeva Z), and two evaluated chest X-ray and HRCT (Köktürk N, Türktaş H). The observers evaluating the lung US and US-E results are pulmonary specialists with several years of LUS interpretation experience. Again, all chest X-ray and HRCT interpreters are well- experienced pulmonary specialists and exceptionally qualified in diagnosing ILDs. The specialists evaluating lung US and US-E were blind to the chest X-ray and HRCT results. According to ultrasound findings, patients were classified as “ILD compatible” and “ILD incompatible”. Similarly, tomography sections and chest radiography were evaluated as “ILD compatible” and “ILD incompatible” independent of US and clinical findings.

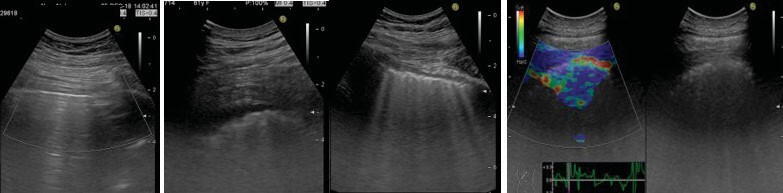

**Figure 1.** Samples from lung ultrasonograpghy examination and elastography.

Regular pleural line and A lines **(A)**. Pleural effusion **(B)**. B lines **(C).** Irregular thick pleura, B lines and blue colour dominant elas- tograpghy **(D)**.

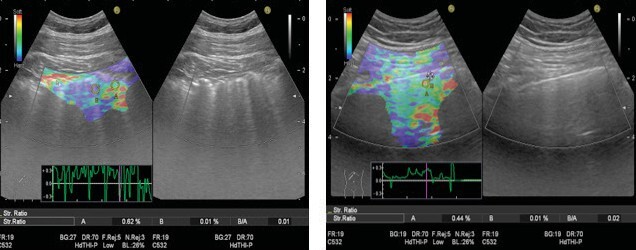

**Figure 2.** Strain ratio evaluation (Strain ratio-B/A, circles indicated ROI; ROI a was selected from the green-red colored area, and ROI B was selected from the blue-colored area). Irreguler pleural line, B line, and blue color predominant elastograpghy **(A)**. Regular pleural line and green color predominant elastography **(B)**.

### Statistical Analysis

In the literature, the effect size of the total number of B-lines for interstitial lung disease in systemic sclerosis was found to be 1.12, which indicates a large effect size (23). In this study, for the association between B-line dominance (presence and absence of B-line predominance) and the status of ILD (presence of ILD and absence of ILD), the effect size was considered large and taken as w= 0.5. For the statistically significant association for a confidence level (1-alpha) of 0.95, achieving 95% power to detect an effect size (w) of 0.5, the minimum required sample size would be 52. The sample size was calculated by using G-Power version 3.1 (24).

The study used descriptive statistics, normality tests, and categorical variables to assess the differences between elastography subgroups. Normality was assessed using Shapiro-Wilk tests, histograms, box lines, and Q-Q plots. Normality differences were compared using Independent Samples t-tests, Mann- Whitney U tests, and Chi-Square or Fisher’s exact tests. Non-parametric Kruskal-Wallis variance analysis examined the relationship between the elastogram color scale, Warrick Score, number of B lines, 6-minute walking test, and pulmonary function test parameters. Computed tomography was used as the gold standard, and the performance of computed tomography and US and chest X-ray results were evaluated with accuracy, sensitivity, specificity, and negative and positive predictive values. Significance level was set at 0.05. Statistical analyses were conducted using IBM SPSS version 23.

## RESULTS

Overall, 109 participants were included in this study. Fifty-five patients were referred to as the “ILD group” and 54 as the ‘‘control group’’. Mean age of the par- ticipants was 62 ± 14 years old. Demographic char- acteristics, primary diagnoses, and concomitant dis- eases of both groups are summarized in Table 2.

Mean 6-minute walking test distance was determined as 341 ± 150 meters in the ILD group; however, it was not evaluated in the control group.

Chest X-ray and tomography findings, pulmonary function tests, arterial blood gas analysis, and pulmonary artery pressure (PAP) according to echocardiography results were compared and summarized in Table 3. The most common pathological radiologic sign was pleural thickening and irregularity in the ILD group (87%) and solitary pulmonary nodule (63%) in the control group.

According to the US examination, most B-lines (77%) were found in the ILD group, and A-lines (67%) were found in the control group (p= 0.001; p= 0.001, respectively). The total B line count was calculated in both groups. The total B line was 187 ± 94 in the ILD group and 51 ± 64 in the control group (p= 0.001). According to the US-E color distribution, blue color was dominant in the ILD group (74% in the ILD group vs 7% in the control group; p= 0.001), and green color was prevalent in the control group (44% in the control group vs 13%; in ILD group p= 0.001) (Table 3). Mean strain ratio was 0.026 in the ILD group and 0.024 in the control group, and there were

**Table:** 

**Table 2.** Comparison of the general characteristic and demographic variables of ILD and control groups			
	**ILD Group (n= 55)**	**Control Group (n= 54)**	**p**
Age, years mean ± SD	60 ± 13	60 ± 15	0.985
Sex (male), n (%)	29 (53)	25 (46)	0.502
Smoking packets, year (median)	19 (26)	15 (22)	0.323
**Primary diagnosis n (%)**			
Chronic hypersensitivity pneumonia	13 (24)	0 (0)	
Idiopathic pulmonary fibrosis	8 (15)	0 (0)	
Connective tissue disease-related ILD	7 (13)	0 (0)	
Combined pulmonary fibrosis emphysema	5 (9)	0 (0)	
Non-specific interstitial pneumonia	3 (6)	0 (0)	
Pulmonary alveolar proteinosis	3 (6)	0 (0)	
Sarcoidosis	2 (4)	0 (0)	
Drug-related ILD	2 (4)	0 (0)	0.001
Organized pneumonia	2 (4)	0 (0)	
Other ILDs*	10 (18)	0 (0)	
Chronic obstructive pulmonary disease (COPD)	0 (0)	5 (26)	
Asthma	0 (0)	4 (21)	
Pneumonia	0 (0)	8 (15)	
Combined Asthma-COPD	0 (0)	4 (7)	
Pulmonary hypertension	0 (0)	2 (11)	
Pulmonary embolism	0 (0)	4 (7)	
Pleural diseases	0 (0)	1(5)	
**Chronic comorbidities, n (%)**			
Hypertension	16 (29)	19 (35)	0.496
**Connective tissue diseases**	**15 (27)**	**4 (7)**	0.025
Diabetes mellitus	10 (18)	8 (15)	0.636
Chronic obstructive pulmonary disease	6 (11)	13 (24)	0.070
**Coronary artery disease**	**4 (7)**	**13 (24)**	0.016
Home long-term oxygen therapy, n (%)	13 (26)	8 (15)	0.243
*Other ILDs: Pulmonary Langerhans cell histiocytosis (n= 1), respiratory bronchiolitis associated interstitial lung disease (n= 1), occupational lung disease/ILD (n= 1), lymphocytic interstitial pneumonia (n= 1), interstitial pneumonia with autoimmune features (IPAF) (n= 1), eosinophilic granulo- matous polyangiitis (n= 1), eosinophilic pneumonia (n= 1), desquamative interstitial pneumonia (n= 1).

no statistically significant differences between the groups (p= 0.648).

The relationship between HRCT findings and total number of B line was examined in the ILD group. The number of B lines was significantly higher in patients with honeycomb pattern, traction bronchiectasis, and septal thickening on HRCT (p= 0.001) and markedly lower in patients with ground-glass opacities (p= 0.033, r= -0.287). No statistically significant correlation was found between pleural thickening and the number of B lines (p= 0.113) (Table 4).

A positive correlation and statistically significant relationship were found between the Warrick score, which helps to determine the severity of the disease, and the number of B-lines detected in the US in ILD patients (p= 0.001, r= 0.550) (Figure 3).

The relationship of US-E color distribution with Warrick score, respiratory function test values, diffusion (DLCO), and 6-minute walking test results were also examined (Table 5). Warrick score, total B

line number, pleural thickness, and FEV1/FVC ratio were significantly higher, and DLCO values were

significantly lower in patients with blue color predominance, which shows tissue stiffness (Table 5).

**Table:** 

**Table 3.** Comparison of the laboratory and radiological findings of ILD and control groups			
	**ILD Group (n= 55)**	**Control Group (n= 54)**	**p**
Chest X-ray (%)			
**Reticular pattern**	**48 (87)**	**8 (15)**	**0.001**
**Nodular pattern**	**23 (42)**	**8 (15)**	**0.002**
**Reticulonodular pattern**	**20 (36)**	**4 (8)**	**0.001**
**Pleural effusion**	**4 (7)**	**16 (30)**	**0.002**
**Pleural thickening**	**16 (29)**	**5 (9)**	**0.010**
Loss of lung volume	36 (66)	29 (55)	0.254
Elevation of diaphragm	30 (55)	23 (43)	0.247
**Hilar lymphadenopathy**	**7 (13)**	**19 (36)**	**0.005**
Hyperinflation	2 (4)	3 (6)	0.963
Opacity	13 (23)	19 (36)	0.165
HRCT, n (%)			
**Pleural thickening & irregularity**	**46 (84)**	**24 (59)**	**0.006**
**Septal thickening**	**41 (75)**	**9 (22)**	**0.001**
**Ground glass opacity**	**33 (60)**	**14 (34)**	**0.012**
**Traction bronchiectasis**	**30 (55)**	**3 (7)**	**0.001**
Solitary pulmonary nodule	27 (49)	26 (63)	0.163
**Honey-comb pattern**	**24 (44)**	**0 (0)**	**0.001**
**Cysts**	**19 (35)**	**2 (5)**	**0.001**
Mosaic attenuation	15 (27)	13 (32)	0.636
Emphysema	**7 (13)**	**16 (39)**	**0.003**
Lung consolidation	6 (11)	6 (15)	0.585
Bronchiectasis	6 (11)	10 (24)	0.080
Nodular pattern	4 (7)	3 (7)	0.993
**Lung mass**	**2 (4)**	**8 (20)**	**0.012**
Ultrasound findings, n (%)			
**A line predominance**	**6 (11)**	**36 (67)**	**0.001**
**B line predominance**	**42 (77)**	**11 (20)**	**0.001**
**Pleural thickening, n (%)**	**47 (86)**	**12 (22)**	**0.001**
Focal thickening	25 (46)	12 (22)	
Diffuse thickening	21 (39)	0 (0)	
**Pleural thickening, mm**	**1.59 ± 0.61**	**1 ± 0.23**	**0.001**
Consolidation	5 (9)	7 (13)	0.741
Pleural effusion	8 (15)	16 (30)	0.057
US-E color scale, n (%)			
**Green predominance**	**7 (13)**	**24 (44)**	**0.001**
**Blue predominance**	**40 (74)**	**4 (7)**	**0.001**
**Blue-green equality**	7 (13)	**21 (39)**	**0.001**
Red predominance	0 (0)	1 (2)	-
Non-specific color	0 (0)	4 (8)	-
Pulmonary function test			
FVC, cc	2454 ± 995	2225 ± 747	0.207
FVC (%)	80 ± 24	80 ± 26	0.920
**FEV1/FVC**	**82 ± 8**	**67 ± 18**	**0.001**
TLC, cc	3832 ± 1034	4339 ± 1469	0.248
**TLC (%)**	**71 ± 17**	**85 ± 18**	**0.029**
**DLCO (%)**	**64 ± 20**	**80 ± 21**	**0.010**
DLCO/VA (%)	77 ± 18	84 ± 17	0,231
Arterial blood analysis			
PaO2, mmHg	75 ± 19	70 ± 22	0.249
PaCO2, mmHg	35 ± 7	38 ± 7	0.071
Oxygen saturation (%)	94 ± 5	93 ± 5	0.191
Echocardiography			
PAP, mmHg	33 ± 18	40 ± 16	0.149
*FVC: Forced vital capacity, FEV1: Forced expiratory volume, TLC: Total lung capacity, DLCO: Carbon monoxide diffusion capacity, VA: Alveolar volume, PAP: Pulmonary arterial pressure.			

**Table:** 

**Table 4.** Evaluation of ultrasonographic total number of B lines according to HRCT findings in the ILD group			
**HRCT Results**	**Total B Lines (n= 55) mean ± SD**	**p**	**R**
Honeycomb pattern			
Presence (+)	238 ± 66	**0.001**	**0.580**
Absence (-)	142 ± 91		
Traction Bronchiectasis			
Presence (+)	232 ± 77	**0.001**	**0.573**
Absence (-)	127 ± 80		
Septal thickening			
Presence (+)	214 ± 81	**0.001**	**0.569**
Absence (-)	99 ± 72		
Pleural thickening			
Presence (+)	192 ± 90	0.113	-
Absence (-)	209 ± 86		
Emphysema			
Presence (+)	185 ± 97	0.790	-
Absence (-)	183 ± 94		
Bronchiectasis			
Presence (+)	179 ± 11	0.399	-
Absence (-)	183 ± 96		
Consolidation			
Presence (+)	167 ± 54	0.845	-
Absence (-)	185 ± 97		
Ground glass opacity			
Presence (+)	165 ± 96	**0.033**	**-0.287**
Absence (-)	209 ± 86		
Mosaic attenuation			
Presence (+)	148 ± 77	0.159	-
Absence (-)	198 ± 97		
Nodules			
Presence (+)	103 ± 98	0.130	-
Absence (-)	188 ± 92

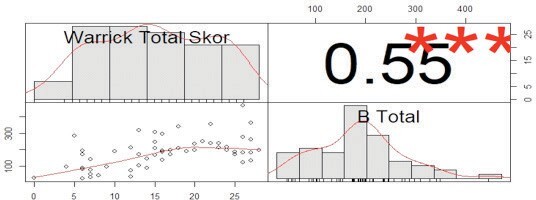

**Figure 3.** Scatter and correlation graph between Warrick's total score and total B line numbers [Warrick score (mean ± SD 16 ± 7, total number of B (mean ± SD) 187 ± 94].

**Table:** 

**Table 5.** Warrick score, the total number of B lines, 6-min walking test results, pulmonary function test, DLCO, and pleural thick- ness according to US-E color scale
**US-E Color Scale**
**Green Blue Blue= Green** ** (mean ± SD) (mean ± SD) (mean ± SD) Red Non-specific p **
Warrick score 11 ± 6 **18 ± 7** 12 ± 7 0 0 **0.013** 6-min WT (m) 322 ± 187 333 ± 158 295 ± 141 207 0.852 FVC (%) 85 ± 25 77 ± 25 81 ± 25 89 90 0.819 FEV1/FVC (%) 72 ± 16 **82 ± 11** 68 ± 18 85 50 **0.001** TLC (%) 93 ± 21 74 ± 16 75 ± 19 0 0 0.090 DLCO (%) 86 ± 15 **66 ± 22** 64 ± 20 - - **0.021** DLCO/VA (%) 86 ± 15 77 ± 17 79 ± 22 - - 0.398 Total B lines 63 ± 69 **197 ± 102** 103 ± 83 61 46 + 30 **0.001** Pleural thickness, mm 1.1 ± 0.5 **1.6 ± 0.5** 1.1 ± 0.3 0.8 1 + 0.12 **0.001**
*FVC: Forced vital capacity, FEV1: Forced expiratory volume, TLC: Total lung capacity, DLCO: Carbon monoxide diffusion capacity, VA: Alveolar volume, 6-min WT: 6-minute walking test.

**Table:** 

**Table 6.** Sensitivity, specificity, accuracy, and predictive values of imaging methods in ILD diagnosis					
	**Accuracy %**	**Sensitivity %**	**Specificity %**	**Negative Predictive Value %**	**Positive Predictive Value %**
US	0.69	0.80	0.60	0.73	0.66
Chest X-ray	0.78	0.67	0.89	0.73	0.86
Chest X-ray & US	0.74	0.60	0.89	0.69	0.85
HRCT	0.84	0.84	0.84	0.78	0.88

According to HRCT, which is accepted as the gold standard imaging method in diagnosing ILD, the diagnostic value of US and chest X-ray was evaluated (Table 6). Lung US diagnosed the ILDs with 69% accuracy, 80% sensitivity and 60% specificity. Combined with a chest X-ray, the diagnosis was with 74% accuracy, 60% sensitivity, and 89% specificity.

## DISCUSSION

In this research, we investigated the role of US and US-E in diagnosing ILDs. With lung US, B lines were found to be more prominent in ILD patients, and a positive correlation was identified between total B lines and the severity of ILDs, which was evaluated with the Warrick score. Honeycomb pattern, traction bronchiectasis, and septal thickening in HRCT correlated positively with the total number of B lines. Since the control group consisted of patients with other lung pathologies rather than ILD, A lines were detected predominantly. These results were consistent with several studies evaluating lung US in ILDs (25- 29). In a meta-analysis, there was a high positive correlation between B lines and the Warrick score

(correlation co-efficient: 0.783; p-< 1 × 10-9), which supports our study (30).

Another remarkable finding in our study is identifying significantly lower B lines in patients with ground- glass opacities (p= 0.033, r= -0.287). Like us, Davidsen JR et al. have reported that although there were ground glass opacities in HRCT, there were no findings in the US in cystic lung disease patients (31). However, Hasan Ali A et al. have shown higher B lines in the presence of ground glass opacities in HRCT than fibrosis (25). The results in the literature are contradictory, and this situation might suggest that the use of US may underestimate the diagnosis, especially during acute exacerbations and early stages of ILDs presenting with ground-glass opacities. However, the use of US can also identify progression to fibrosis and may be used to assess disease severity. Further evidence is necessary to clarify this issue.

We also identified pleural thickening and irregularity as the second common finding of lung US in ILD patients. Pinal-Fernandez I. et al. have emphasized that pleural irregularity is more effective than the B

line in detecting ILD (32). Pleural thickness has been identified differently in various studies. Some researchers have accepted pleural thickness >3 mm, and others have accepted >1 mm (27,33). In our study, regular, healthy pleura thickness was found to be ≤ 1 mm, and it was confirmed with HRCT results. As a limitation, we did not use a linear transducer for the evaluation of pleural thickening and irregularity, and since we performed a comprehensive US assessment from 50 intercostal spaces, it would be more time- consuming to evaluate by both linear transducer and microconvex probe for the same patient.

Transthoracic surface wave elastography has been used in many studies to evaluate ILD, and a significant difference in surface wave velocity was reported among ILD patients (18-21). A study that compared surface wave elastography in healthy and ILD patients showed that surface wave elastography had 92% sensitivity and 89% specificity for diagnosing ILDs

(18). Our study used the strain elastography method since our ultrasound device and elastography probe were incompatible with surface wave elastography.

The strain imaging technique applies an average pressure to the liver, breast, and thyroid tissue with the ultrasound probe (34). Since the lungs are not superficial organs located in the thorax, it is technically challenging to perform strain elastography by applying pressure from the intercostal distance. We used a micro convex probe to provide the correct and more accessible accommodation of the probe to the intercostal area. In order to obtain a strain elastography image, low pressure from the intercostal distance was applied and/or patients were asked to take several deep breathings. We observed that the more accessible elastogram image was obtained in the control group compared to the ILD group. Our observation suggests that the lungs in the control group were more elastic than the ILD group. Soft, non-fibrotic lungs lead to easy imaging by deep breathing and getting closer to the rib cage. We observed another problem in obese patients during US-E imaging. Increased subcutaneous adipose tissue prevented imaging in obese patients, especially female ones. Besides, we had seven patients in our ILD group who had connective tissue disease-related ILDs. Three of them had systemic sclerosis, two of them had rheumatoid arthritis, and two of them had Sjögren syndrome. The increase in skin thickness in systemic sclerosis patients might be considered a confounding problem when evaluating strain

elastography of the lung. Some studies assessed skin thickness and fibrosis with US-E in systemic sclerosis (35,36). However, we could not find any information in the literature about whether this skin thickness in scleroderma patients may interfere with the evaluation of lung elastography. Our patient number with systemic sclerosis was not enough to evaluate this factor, and this point may be assessed in the future with prospective studies. All the difficulties mentioned above might lead to our result of a non-significant relationship in strain ratios between ILD and the control group.

Although no difference was identified among strain ratios, US-E color intensity differed between ILD and control groups. Blue color, which indicates stiffer tissue, was more prominent in the ILD group (74%, p= 0.001). However, a green color, which means soft tissue, was more pronounced in the control group (%44, p= 0.001). In the literature, strain elastography has been applied to evaluate peripheral pulmonary lesions; however, lesions scored according to color scale scoring (37,38), not color intensity (17). In our study, a color scale scoring system was not used, but the results suggested that color intensity, especially blue color dominancy, could indicate possible lung fibrosis.

Our study identified that lung US had 69% accuracy and 80% sensitivity compared to HRCT, but its specificity was 60% in diagnosing ILD. Performing chest X-rays with the US had 74% accuracy, 60% sensitivity, and 89% specificity in diagnosing ILD. Vizioli L et al. have shown chest X-ray is specific (91%; 95% CI 80-100) but not sensitive (48%; 95% CI 28-67) in ILD, and US had a high sensitivity (92%; 95% CI 84-99), but low specificity (79%; 95% CI 69-90%) (26). They have suggested combining chest X-ray and lung US may reduce the need for HRCT

(26). In a meta-analysis of 349 connective tissue disease-related ILD patients, the sensitivity and specificity of lung US has been found to be 91.5% and 81.3%, respectively (30). In 147 patients with rheumatoid arthritis lung involvement, the sensitivity of US was 87.0%, and the specificity was 72.7% (39). One of the reasons for the different specificity of US results in our study could be related to our study population. Our ILD patient group consisted of whole people with suspected ILDs, not a specific disease subgroup (such as connective tissue disease, RA) as in other studies. Subjective interpretation of color distribution in elastography and not using any

countable scoring system are other limitations of our research.

## CONCLUSION

In conclusion, conventional lung US and US-E are inferior to gold standard HRCT in diagnosing ILDs. However, they might be helpful to support the diagnosis and assess the severity. They might be accepted as promising, novel, non-invasive, alternative tools, especially when combined with chest X-rays. Their role in the early diagnosis of ILDs should be clarified within the near future with the advancement of technology and with new, more comprehensive studies.

**Ethical Committee Approval:** This study was approved by the Gazi University Faculty of Medicine Clinical Research Ethics Committee (Decision no: 149, Date: 26.02.2018).

### CONFLICT of INTEREST

The authors declare that they have no conflict of interest.

## AUTHORSHIP CONTRIBUTIONS

Concept/Design: MA, ZA, EÖ Analysis/Interpretation: MA, ZA, NK, HŞT Data acqusition: NYD, MA, ZA

Writing: EÖ, MA, ZA Clinical Revision: MA

Final Approval: All of authors

## REFERENCES

1. *
  Cottin V, Hirani NA, Hotchkin DL, Nambiar AM, Ogura T, Otaola M, et al. Presentation, diagnosis and clinical course of the spectrum of progressive-fibrosing interstitial lung diseases. Eur Respir Rev 2018; 27(150): 180076. https://doi.org/10.1183/16000617.0076-2018*
2. *
  Raghu G, Remy-Jardin M, Myers JL, Richeldi L, Ryerson CJ, Lederer DJ, et al. American Thoracic Society, European Respiratory Society, Japanese Respiratory Society, and Latin American Thoracic Society. Diagnosis of Idiopathic Pulmonary Fibrosis. An Official ATS/ERS/JRS/ALAT Clinical Practice Guideline. Am J Respir Crit Care Med 2018; 198(5): e44-e68.
  *
3. *
  Travis WD, Costabel U, Hansell DM, King TE Jr, Lynch DA, Nicholson AG, et al. An official American Thoracic Society/European Respiratory Society statement: Update of the international multidisciplinary classification of the idiopathic interstitial pneumonia. Am J Respir Crit Care Med 2013; 188: 733-48. https://doi.org/10.1164/rccm.201308-1483ST*
4. *
  Marini TJ, Rubens DJ, Zhao YT, Weis J, O’Connor TP, Novak WH, et al. Lung ultrasound: The essentials. Radiol Cardiothorac Imaging 2021; 3(2): e200564. https://doi.org/10.1148/ryct.2021200564*
5. *
  Romero Romero B, Vollmer Torrubiano I, Martín Juan J, Heili Frades S, Pérez Pallares J, Pajares Ruiz V, et al. Ultrasound in the study of thoracic diseases: Innovative aspects. Arch Bronconeumol 2024; 60(1): 33-43. https://doi.org/10.1016/j.arbres.2023.10.009*
6. *
  Santos-Moreno P, Linares-Contreras MF, Rodríguez-Vargas GS, Rodríguez-Linares P, Mata-Hurtado A, Ibatá L, et al. Usefulness of lung ultrasound as a method for early diag- nosis of interstitial lung disease in patients with rheuma- toid arthritis. Open Access Rheumatol 2024; 16: 9-20. https://doi.org/10.2147/OARRR.S441720*
7. *
  Laursen CB, Clive A, Hallifax R, Pietersen PI, Asciak R, Davidsen JR, et al. European Respiratory Society state- ment on thoracic ultrasound. Eur Respir J 2021; 57(3): 2001519. https://doi.org/10.1183/13993003.01519-*
8. *
  Manolescu D, Davidescu L, Traila D, Oancea C, Tudorache V. The reliability of lung ultrasound in assessment of idio- pathic pulmonary fibrosis. Clin Interv Aging 2018; 13: 437-49. https://doi.org/10.2147/CIA.S156615*
9. *
  Soldati G, Demi M, Inchingolo R, Smargiassi A, Demi L. On the physical basis of pulmonary sonographic intersti- tial syndrome. J Ultrasound Med 2016; 35: 2075-86. https://doi.org/10.7863/ultra.15.08023*
10. *
  Gargani L, Bruni C, Romei C, Frumento P, Moreo A, Agoston G, et al. Prognostic value of lung ultrasound B lines in systemic sclerosis. Chest 2020; 158(4): 1515-25. https://doi.org/10.1016/j.chest.2020.03.075*
11. *
  Lim CK, Chung CL, Lin YT, Chang CH, Lai YC, Wang HC, et al. Transthoracic ultrasound elastography in pulmonary lesions and diseases. Ultrasound Med Biol 2017; 43(1): 145-52. https://doi.org/10.1016/j.ultrasmed-bio.2016.08.028*
12. *
  Sigrist RM, Liau J, El Kaffas A, Chammas MC, Willmann JK. Ultrasound elastography: Review of techniques and clini- cal applications. Theranostics 2017; 7(5): 1303-29. https://doi.org/10.7150/thno.18650*
13. *
  Barr RG. Elastography in clinical practice. Radiologic Clinics 2014; 52(6): 1145-62. https://doi.org/10.1016/j.rcl.2014.07.002*
14. *
  Doyley MM, KJ Parker. Elastography: General principles and clinical applications. Ultrasound Clinics 2014; 9(1): 1-11. https://doi.org/10.1016/j.cult.2013.09.006*
15. *
  Leong SS, Wong JHD, Ng KH. Ultrasound based elastogra- phy: Principles and clinical applications. Med Phys Int 2023; 11(1): 85-90.
  *
16. *
  Ozgokce M, Yavuz A, Akbudak I, Durmaz F, Uney I, Aydin Y, et al. Usability of transthoracic shear wave elastography in differentiation of subpleural solid masses. Ultrasound Q 2018; 34(4): 233-7. https://doi.org/10.1097/RUQ.0000000000000374*
17. *
  Wei H, Lu Y, Ji Q, Zhou H, Zhou X. The application of conventional ultrasound and transthoracic ultrasound elastography in evaluating peripheral pulmonary lesions. Exp Ther Med 2018; 16(2): 1203-8. https://doi.org/10.3892/etm.2018.6335*
18. *
  Zhou B, Bartholmai BJ, Kalra S, Osborn TG, Zhang X. Lung US surface wave elastography in interstitial lung disease staging. Radiology 2019; 291(2): 479-84. https://doi.org/10.1148/radiol.2019181729*
19. *
  Clay R, Bartholmai BJ, Zhou B, Karwoski R, Peikert T, Osborn T, et al. Assessment of interstitial lung disease using lung ultrasound surface wave elastography: A novel technique with clinicoradiologic correlates. J Thorac Imaging 2019; 34(5): 313-9. https://doi.org/10.1097/RTI.0000000000000334*
20. *
  Zhang X, Zhou B, Osborn T, Bartholmai B, Kalra S. Lung ultrasound surface wave elastography for assessing inter- stitial lung disease. IEEE Trans Biomed Eng 2019; 66(5): 1346-52. https://doi.org/10.1109/TBME.2018.2872907*
21. *
  Zhang X, Zhou B, Bartholmai B, Kalra S, Osborn T. A quan- titative method for measuring the changes of lung surface wave speed for assessing disease progression of interstitial lung disease. Ultrasound Med Biol 2019; 45(3): 741-8. https://doi.org/10.1016/j.ultrasmedbio.2018.11.003*
22. *
  Warrick JH, Bhalla M, Schabel SI, Silver RM. High resolu- tion computed tomography in early scleroderma lung disease. J Rheumatol 1991; 18(10): 1520-8.
  *
23. *
  Barskova T, Gargani L, Guiducci S, Randone SB, Bruni C, Carnesecchi G, et al. Lung ultrasound for the screening of interstitial lung disease in very early systemic sclerosis. Ann Rheum Dis 2013; 72(3): 390-5. https://doi.org/10.1136/annrheumdis-2011-201072*
24. *
  Faul F, Erdfelder E, Buchner A, Lang AG. Statistical power analyses using G*Power 3.1: Tests for correlation and regression analyses. Behav Res Methods 2009; 41(4): 1149-60. https://doi.org/10.3758/BRM.41.4.1149*
25. *
  Hasan AA, Makhlouf HA. B-lines: Transthoracic chest ultrasound signs helpful in the assessment of interstitial lung diseases. Ann Thorac Med 2014; 9(2): 99-103. https://doi.org/10.4103/1817-1737.128856*
26. *
  Vizioli L, Ciccarese F, Forti P, Chiesa AM, Giovagnoli M, Mughetti M, et al. Integrated use of lung ultrasound and chest X-ray to detect interstitial lung disease. Respiration 2017; 93(1): 15-22. https://doi.org/10.1159/000452225*
27. *
  Sayed SS, Agmy GM, Said AF, Kasem AH. Assessment of transthoracic sonography in patients with interstitial lung diseases. Egypt J Bronchol 2016; 10: 105-12. https://doi.org/10.4103/1687-8426.184375*
28. *
  Dubinsky TJ, Shah H, Sonneborn R, Hippe DS. Correlation of B-Lines on ultrasonography with interstitial lung disease on chest radiography and CT imaging. Chest 2017; 152(5): 990-8. https://doi.org/10.1016/j.chest.2017.05.003 *
29. *
  Man MA, Dantes E, Domokos Hancu B, Bondor CI, Ruscovan A, Parau A, et al. Correlation between transtho- racic lung ultrasound score and HRCT features in patients with interstitial lung diseases. J Clin Med 2019; 8(8): 1199. https://doi.org/10.3390/jcm8081199*
30. *
  Song G, Bae SC, Lee YH. Diagnostic accuracy of lung ultrasound for interstitial lung disease in patients with connective tissue diseases: A meta-analysis. Clin Exp Rheumatol 2016; 34(1): 11-6.
  *
31. *
  Davidsen JR, Bendstrup E, Henriksen DP, Graumann O, Laursen CB. Lung ultrasound has limited diagnostic value in rare cystic lung diseases: A cross-sectional study. Eur Clin Respir J 2017; 4(1): 1330111. https://doi.org/10.1080/20018525.2017.1330111*
32. *
  Pinal-Fernandez I, Pallisa-Nuñez E, Selva-O’Callaghan A, Castella-Fierro E, Simeon-Aznar CP, Fonollosa-Pla V, et al. Pleural irregularity, a new ultrasound sign for the study of interstitial lung disease in systemic sclerosis and antisyn- thetase syndrome. Clin Exp Rheumatol 2015; 33(4 Suppl 91): S136-41.
  *
33. *
  Sperandeo M, Varriale A, Sperandeo G, Filabozzi P, Piattelli ML, Carnevale V, et al. Transthoracic ultrasound in the evaluation of pulmonary fibrosis: Our experience. Ultrasound Med Biol 2009; 35(5): 723-9. https://doi.org/10.1016/j.ultrasmedbio.2008.10.009*
34. *
  Dietrich CF, Barr RG, Farrokh A, Dighe M, Hocke M, Jenssen C, et al. Strain elastography - How to do it? Ultrasound Int Open 2017; 3(4): E137-49. https://doi.org/10.1055/s-0043-119412*
35. *
  Yang Y, Yan F, Wang L, Xiang X, Tang Y, Li Q, et al. Quantification of skin stiffness in patients with systemic sclerosis using real time shear wave elastography: A pre- liminary study. Clin Exp Rheumatol 2018; 36 (suppl 113):118-25.
  *
36. *
  Sobolewski P, Maślińska M, Zakrzewski J, Paluch Ł, Szymańska E, Walecka I. Applicability of shear wave elas- tography for the evaluation of skin strain in systemic scle- rosis. Rheumatol Int 2020; 40: 737-45. https://doi.org/10.1007/s00296-020-04539-y*
37. *
  Hahn S, Lee YH, Lee SH, Suh JS. Value of the strain ratio on ultrasonic elastography for differentiation of benign and malignant tumors. J Ultrasound Med 2017; 36: 121-7. https://doi.org/10.7863/ultra.16.01054*
38. *
  Itoh A, Ueno E, Tohno E, Kamma H, Takahashi H, Shiina T et al. Breast disease: Clinical application of US elastogra- phy for diagnosis. Radiology 2006; 239(2): 341-50. https://doi.org/10.1148/radiol.2391041676*
39. *
  Antivalle M, Chevallard M, Battellino M, Ditto MC, Varisco V, Rigamonti F, et al. Lung ultrasound screening for intersti- tial lung disease in rheumatoid arthritis. Comparison with usual detection algorithms in clinical practice. 2014 ACR/ ARHP Annual Meeting, Abstract No: 2976. Arthritis Rheumatol 2014; 66: S1301.
  *

